# Targeting Sporadic and Neurofibromatosis Type 1 (NF1) Related Refractory Malignant Peripheral Nerve Sheath Tumors (MPNST) in a Phase II Study of Everolimus in Combination with Bevacizumab (SARC016)

**DOI:** 10.1155/2019/7656747

**Published:** 2019-07-24

**Authors:** Brigitte C. Widemann, Yao Lu, Denise Reinke, Scott H. Okuno, Christian F. Meyer, Gregory M. Cote, Rashmi Chugh, Mohammed M. Milhem, Angela C. Hirbe, AeRang Kim, Brian Turpin, Joseph G. Pressey, Eva Dombi, Nalini Jayaprakash, Lee J. Helman, Ndidi Onwudiwe, Karen Cichowski, John P. Perentesis

**Affiliations:** ^1^National Cancer Institute, Center for Cancer Research, Pediatric Oncology Branch, 10 Center Drive, Building 10, Room 1-3752, Bethesda, MD 20892, USA; ^2^SARC Statistics, Weill Cornell Medicine Healthcare and Policy Research, 402 East 67th Street, New York, NY 10065, USA; ^3^SARC, 24 Frank Lloyd Wright Drive, Ann Arbor, MI 48105, USA; ^4^Mayo Clinic, 200 First St, SW, Rochester, MN 55905, USA; ^5^Johns Hopkins Hospital, 1650 Orleans St., CRB1/Room G89, Baltimore, MD 21231, USA; ^6^Massachusetts General Hospital Cancer Center, Harvard Medical School, 10 North Grove Street, POB-2, Boston, MA 02114, USA; ^7^University of Michigan, 1500 E. Medical Center Dr, SPC 5912, Ann Arbor, MI 48109, USA; ^8^University of Iowa, 200 Hawkins Drive, C32 GH, Iowa, IA 52242, USA; ^9^Washington University of St. Louis, 660 S, Euclid Ave, St. Louis, MO 63110, USA; ^10^Children's National Medical Center, 111 Michigan Ave, NW, Washington, DC 20010, USA; ^11^Cincinnati Children's Hospital, 3333 Burnet Ave, Cincinnati, OH 45229, USA; ^12^Cincinnati Children's Hospital Medical Center, University of Alabama, 1600 7th Avenue South, Lowder 512, Birmingham, AL 35233, USA; ^13^Brigham and Women's Hospital, 75 Francis Street, Boston, MA 02115, USA

## Abstract

**Purpose:**

There are no known effective medical treatments for refractory MPNST. Inactivation of the NF1 tumor suppressor in MPNST results in upregulation of mTOR (mammalian target of rapamycin) signaling and angiogenesis, which contributes to disease progression. We conducted a phase II study for patients (pts) with refractory MPNST combining everolimus (10 mg PO once daily) with bevacizumab (10 mg/kg IV every 2 weeks) to determine the clinical benefit rate (CBR) (complete response, partial response (PR), or stable disease (SD) ≥ 4 months).

**Patients and Methods:**

Patients ≥18 years old with chemotherapy refractory sporadic or NF1 MPNST were eligible. Tumor response was assessed after every 2 cycles (the WHO criteria). A two-stage design targeting a 25% CBR was used: if ≥ 1/15 pts in stage 1 responded, enrollment would be expanded by 10 pts, and if ≥ 4/25 patients had clinical benefit, the combination would be considered active.

**Results:**

Twenty-five pts, 17 with NF1 and 8 with sporadic MPNST, enrolled. One of 15 pts in stage 1 had clinical benefit. Of 10 additional pts enrolled, 2 had clinical benefit. The median number of completed cycles was 3 (range 1–16). Adverse events were similar to those known for this combination.

**Conclusion:**

With a CBR of 12% (3/25), the combination of everolimus and bevacizumab did not reach the study's target response rate and is not considered active in refractory MPNST.

## 1. Introduction

Malignant peripheral nerve sheath tumors (MPNST) are rare and clinically aggressive soft tissue sarcomas that occur with greater incidence in individuals with neurofibromatosis type 1 (NF1) [1, 2]. Complete surgical resection is required for cure. Response to cytotoxic chemotherapy used to treat other soft tissue sarcomas is poor [3]. To date, no phase II trials with targeted therapies have resulted in clinical benefit as demonstrated by tumor shrinkage or improvement in progression-free survival [4].

In an *Nf1*/*p53*-mutant MPNST model, the Cichowski group identified that the mammalian target of rapamycin (mTOR) is hyperactive and that the mTOR inhibitor sirolimus substantially delayed tumor growth [5]. Angiogenesis contributes to progression of MPNST, and in this mouse model, development of resistance was associated with revascularization and upregulation of the vascular endothelial growth factor. The combination of sunitinib, a multitargeted kinase inhibitor, which in part, mediates antitumor activity by inhibition of angiogenesis, with sirolimus resulted in prolongation of survival compared to treatment with either agent alone (Cichowski lab, unpublished data).

As angiogenesis appears critical to the development of resistance to treatment with sirolimus *in vivo* and the tolerability of sunitinib with an mTOR inhibitor raised concerns, we elected to develop a phase II trial combining the mTOR inhibitor everolimus with the recombinant humanized anti-VEGF monoclonal antibody bevacizumab to evaluate the clinical activity of this combination in patients with sporadic or NF1-associated MPNST. At the time of development of our trial, the tolerable doses and safety of this combination had been established [6].

## 2. Materials and Methods

### 2.1. Patient Population

Eligibility criteria were as follows: patients aged ≥18 years with histologically confirmed unresectable, refractory, or metastatic high-grade NF1 or sporadic MPNST; presence of measurable disease; progressive disease after ≥1 prior cytotoxic chemotherapy (unless the patient refused chemotherapy or chemotherapy was felt not to be in the best interest of the patient by the treating physician); Eastern Cooperative Oncology Group (ECOG) performance status of 0 to 2; adequate bone marrow, liver, and renal function; fasting serum cholesterol ≤300 mg/dL and triglycerides ≤2.5x the upper limit of normal; urine protein creatinine ratio ≤0.5; left ventricular ejection fraction ≥50% in patients who previously received an anthracycline; recovery from toxic effects of prior therapy to ≤grade 1 (CTCAE version 4); a minimum of 3 weeks from prior chemotherapy, 7 days from a biologic agent, 4 weeks from radiation, and 4 weeks from major surgery; willingness to use birth control, and for patients with NF1, documentation of diagnostic criteria [7]. Key exclusion criteria were as follows: patients receiving chronic systemic administration of a corticosteroid or another immunosuppressive agent; presence of severe or uncontrolled medical condition that could affect study participation including brain or leptomeningeal metastases, heart failure, severely impaired lung function, and active or uncontrolled hepatitis; prior treatment with an mTOR inhibitor for MPNST or bevacizumab; concurrent use of anticoagulant drugs at treatment doses, strong CYP3A4 inhibitors, St. John's Wort, grapefruit, or enzyme inducing anticonvulsants.

This multi-institutional trial was coordinated through the Sarcoma Alliance for Research through Collaboration (SARC) and funded by a Department of Defense Clinical Trial Award W81XWH-10-1-0681. The trial was IND exempt. Everolimus was supplied by Novartis and bevacizumab by Genentech. The study was conducted after approval from the Department of Defense Protocol Review, and institutional review boards from all participating sites and all patients provided written informed consent before participating. The trial was registered with ClinicalTrials.gov (NCT01661283).

### 2.2. Study Design

#### 2.2.1. Treatment Overview

Everolimus (2.5, 5, and 10 mg tablets) was administered at 10 mg per dose once daily at the same time on a continuous dosing schedule. Bevacizumab (400 mg vials) was administered at 10 mg/kg as intravenous infusion over 30–90 minutes every 2 weeks (days 1 and 15). One treatment cycle was 28 days. All patients on study received prophylaxis against *Pneumocystis jirovecii* pneumonia using trimethoprim/sulfamethoxazole or inhaled pentamidine. Treatment could continue until a maximum of 2 years and until disease progression or unacceptable toxicity. Adverse events were graded using CTCAEv4 and attributed to either or both study agents. Detailed guidelines were provided for the management of drug-related adverse events such as pneumonitis, hypertension, mucositis, and proteinuria.

For everolimus-related adverse events, up to 2 dose reductions (from 10 mg to 5 mg and then to 2.5 mg per dose) were permitted, provided adverse events resolved within 3 weeks to ≤grade 1. Everolimus was dose reduced for related intolerable adverse events including grade 2 pneumonitis and for grade 3 adverse events (with exception of reversible elevation of alanine aminotransferase (ALT) or aspartate aminotransferase (AST), nausea and vomiting less than 3 days, or grade 3 hyperlipidemia). Everolimus was permanently discontinued for related grade 4 adverse events. For bevacizumab-related adverse events, no dose reductions were permitted, and treatment was permanently discontinued for most grade 3 and all grade 4 related adverse events, and if bevacizumab treatment was interrupted for ≥8 weeks.

### 2.3. Assessments

History and physical examination including vital signs (blood pressure and O_2_ saturation) and laboratory studies including complete blood counts with differential count, fasting glucose, and lipid panel, and comprehensive chemistry panel (creatinine, blood urea nitrogen, albumin, total protein, AST, ALT, total bilirubin, alkaline phosphatase, and uric acid) were performed prior to every treatment cycle. Response evaluation (the WHO criteria) with appropriate imaging studies and evaluation of cardiac function by echocardiogram were performed before every other treatment cycle (3, 5, 7, etc.). Peripheral blood samples were obtained for analysis of VEGF and VEGFR, S6K1 (p70s6 kinase) activity, eIF4E, eIF2*α*, and AKT phosphorylation at baseline and prior to cycles 3 and 5.

### 2.4. Statistical Methods

 A two-stage Simon Minimax phase II design was used to determine the clinical benefit rate(CBR) (number of patients experiencing a complete response (CR), a partial response (PR), or stable disease (SD) for ≥4 months). Confirmation of responses was required. The target clinical benefit rate was 25%, and a clinical benefit rate <5% would be considered uninteresting. Fifteen patients were to enroll on the first stage, and if ≥1 of 15 patients experienced clinical benefit, enrollment would expand to a total of 25 patients. With clinical benefit in ≥4/25 patients, everolimus in combination with bevacizumab would be considered active in that it would be consistent with a 25% clinical benefit rate. Assuming the number of successes is binomially distributed, this design has a one-sided alpha of 5.0% and a power of 90% for detecting a true success probability of at least 25% versus the null hypothesis success rate of 5% or less. Pharmacodynamic endpoints were summarized with descriptive statistics.

## 3. Results

### 3.1. Enrollment Characteristics

Twenty-five eligible patients enrolled on the trial: 15 enrolled on the first stage and 10 on the second stage. Characteristics of all patients and separately for patients with NF1 (*N* = 17) and with sporadic (*N* = 8) MPNST are listed in Table 1. Patients with NF1 MPNST were younger (median age 28 (range, 19, 63) years) than patients with sporadic MPNST (median age 61 (range, 19, 81) years). More females than males enrolled. The most frequent primary tumor location was in the extremities, and only 1 tumor involved the face (parotid gland). While the majority of the primary tumors were initially resected, approximately half had microscopic or macroscopic positive margins and 15 of 20 initially resected tumors recurred locally. At the initial diagnosis, only 14% of patients had metastatic disease, but at enrollment on this trial, 90% had metastatic disease. The majority of patients (92%) had received cytotoxic chemotherapy prior to enrollment (median number of regimens 2, range 0–5), and 84% of patients had received prior radiation therapy. Ninety-two percent of patients had a baseline ECOG of 0 or 1.

### 3.2. Response Evaluation

**Figure 1:**
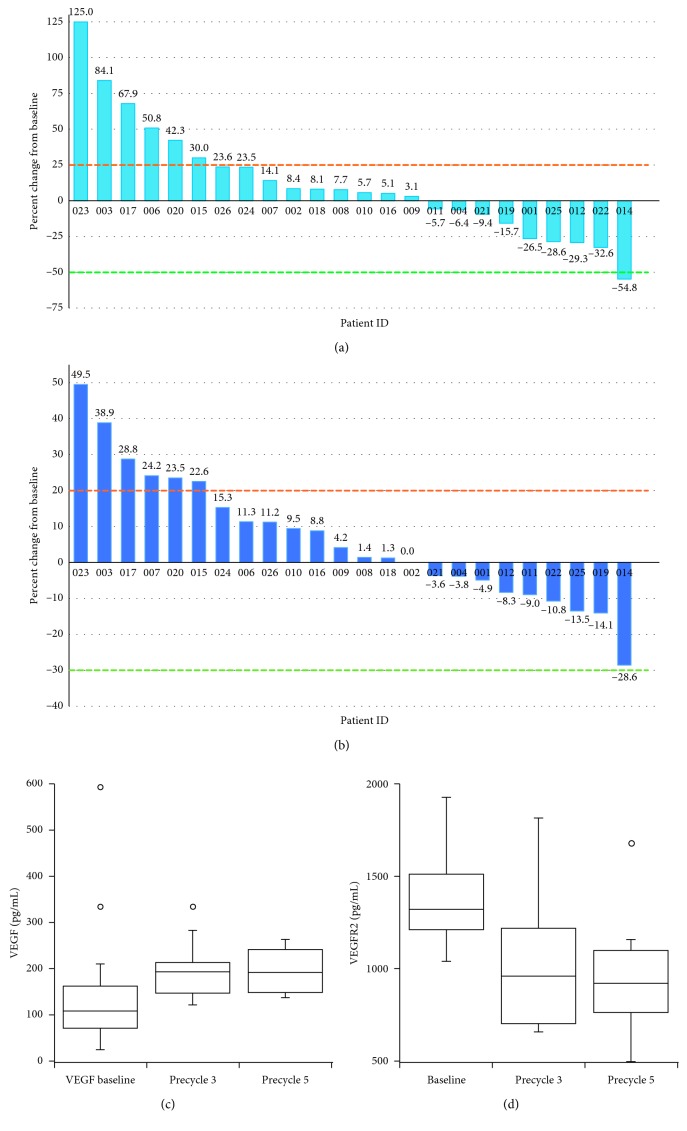
Waterfall plots for best response by the WHO (a) and RECIST (b) and changes in plasma VEGF (c) and VEGFR2 (d) in patients with paired samples at baseline and prior to cycles 3 (*N* = 13) and 5 (*N* = 8).

This study used the WHO criteria [8] to evaluate the response, given that most MPNST are not spherical. Of 15 patients enrolled on the first stage, 1 patient had clinical benefit with SD at the 4-month evaluation (Table 2). This patient had a sporadic MPNST and experienced progressive disease at the post-cycle 6 evaluation. One additional patient had a partial response at the pre-cycle 3 evaluation, which was not confirmed at subsequent restaging. Given that 1 patient had experienced clinical benefit, an additional 10 patients were enrolled on the second stage, of whom 2 experienced clinical benefit with SD as best response. Both patients had NF1 MPNST; one experienced grade 5 intraabdominal hemorrhage during cycle 7, considered possibly related to bevacizumab, and the other patient had stable disease for 16 cycles when disease progressed. The median cycle number in all patients was 3 (range 1–16). Thus, with a total of 3/25 patients with clinical benefit, the target clinical benefit rate was not reached and the combination of everolimus and bevacizumab was considered inactive. In addition to evaluating response by the WHO, as a secondary objective, we compared the WHO response to RECIST response (Table 2). Using RECIST, in the first stage, the best response was clinical benefit with SD in 2 patients, and in the second stage, the best response was clinical benefit with SD in 4 patients. Thus, had our study used RECIST criteria, the desired clinical benefit rate would have been 6/25 patients and the target response rate would have been reached. The waterfall plots (Figures 1(a) and 1(b)) for the WHO and RECIST best response shows that response trends were similar with a substantial number of tumors being stable, followed by tumors clearly growing and one tumor with clear tumor shrinkage.

### 3.3. Adverse Events

The combination of everolimus and bevacizumab was tolerated with adverse events similar to those known for this combination (supplemental Table 1 for all adverse events and all drug-related adverse events). Most adverse events were grade 1 or 2. The most frequent drug-related adverse events were oral mucositis, nausea, vomiting, fatigue, weight loss, anorexia, and hypertension. Grade 3 drug-related adverse events which occurred in more than 1 patient were mucositis (*n* = 5), ALT elevation (*n* = 3), hypophosphatemia (*n* = 3), and fatigue (*n* = 2). Everolimus was dose reduced in 4 patients for grade 1–3 adverse events. Everolimus and bevacizumab treatment were discontinued permanently in 7 patients who experienced grade 1–5 adverse events, not all of which were considered related to study drugs (Table 3).

Reasons for discontinuation of therapy were disease progression (*N* = 19), adverse events (*n* = 4), initiation of systemic treatment with dexamethasone, which was prohibited as concomitant medication (*n* = 1), and physician decision (*n* = 1).

### 3.4. Pharmacodynamic Endpoints

Samples for analysis of VEGF and VEGFR were collected for 23 patients at baseline, 14 patients at the time of the first restaging, and 8 patients at the time of the second restaging. In patients with paired samples, VEGF on treatment increased and VEGFR2 decreased compared to baseline (Figures 1(c) and 1(d)).

## 4. Discussion

This phase II trial was directed at patients with refractory NF1 or sporadic MPNST. Patients enrolled indeed had refractory disease; the majority had prior surgery, chemotherapy, and radiation therapy. While, based on the literature, approximately 50% of MPNST occur in individuals with NF1 [1], this trial enrolled more NF1 MPNST (*N* = 17) compared to sporadic MPNST (*N* = 8). As reported previously, patients with NF1 MPNST were younger (median age 28 years) compared with sporadic MPNST (median age 61 years), consistent with *NF1* being a tumor suppressor [9]. MPNST in NF1 frequently develop in preexisting neurofibromas, which may make surgical removal of MPNST more difficult [1, 4]. However, the majority of NF1 and sporadic MPNST were resected, and the incidence of microscopic and macroscopic positive margins was similar in sporadic and NF1 MPNST. Many tumors recurred locally, and at the time of enrollment, most patients had metastatic and disease underlying the aggressive clinical behavior of MPNST.

The goal of our study was to evaluate whether the combination of the mTOR inhibitor everolimus with the angiogenesis inhibitor bevacizumab would result in a modest clinical benefit rate, which included confirmed partial and complete responses and disease stability for four or more treatment cycles. Our study used the WHO criteria [8] to assess the response, given that most MPNST are nonspherical. In stage 1 and 2 combined, 3/25 patients experienced clinical benefit, all with SD as best response. One of these patients had extended stable disease for over 1 year, which is remarkable for MPNST, most of which are known to progress rapidly. In addition, one patient in the initial stage had a PR at the time of first restaging; however, at the time of restaging after 4 cycles, the patient had PD and this was not counted as a success. Thus, our study did not reach the desired CBR of ≥4/25 and the combination of everolimus and bevacizumab is considered inactive. The WHO criteria define the growth of any target lesion by ≥25% as PD [8]. This is in contrast to RECIST where the sum of the longest diameters of the target lesions is used to determine progression [10]. When we applied RECIST criteria to our study, 6/25 patients would have met the criteria for clinical benefit. Irrespective of the response criteria selected, only a small proportion of patients experienced meaningful disease stabilization. Similar to results in the NF1 MPNST mouse model, we did not observe tumor shrinkage in our trial [5]. In the NF1 mouse model, sirolimus nearly doubled survival. We used duration of SD as an indicator for benefit, and in that light, it may be helpful to reconsider the utility of RECIST versus WHO criteria. The selection of the WHO over RECIST criteria may be most meaningful in upfront trials for the more precise measurement of primary tumors. In the setting of refractory disease where most patients have metastases, which are less complex, the selection of the WHO over RECIST criteria may not provide an advantage. Standardized criteria in future clinical trials which allow for a meaningful comparison of response and disease stability and comparison to mouse preclinical studies will be helpful [11]. In addition, careful comparisons of trial design, pharmacokinetics, and endpoints in preclinical and clinical trials will be helpful to determine whether responses achieved in preclinical studies can be translated to patients [12]. For example, ideally tumors would be measured using similar criteria in preclinical and clinical trials. Assuring that drug exposures are similar in the preclinical and clinical setting would also be helpful for a more meaningful comparison of results. In our study, bevacizumab and everolimus were administered at the single-agent phase II recommended doses. The combination of bevacizumab and everolimus was tolerated with dose reductions of everolimus in 4 patients and discontinuation of bevacizumab in 3 patients for related adverse events. Pharmacodynamic studies for bevacizumab in blood samples were consistent with target inhibition. Due to lack of activity in our trial and previously documented inhibition of targets downstream of mTOR at comparable doses of everolimus, we did not perform pharmacodynamic analyses for mTOR inhibition [13, 14]. A limitation of our study was that tumor biopsies were not collected. Pretreatment and on-treatment tumor biopsies for genomic and metabolomic studies will have utility in assessing mechanisms of response and resistance, thus advancing better therapies for MPNST. These studies are being incorporated in future SARC coordinated trials targeting MPNST, provided biopsies can be obtained safely. Finally, we believe that combinatorial targeted treatment approaches will also have applicability to NF1-related plexiform neurofibromas and atypical neurofibromas.

## 5. Conclusions

While the combination of everolimus and bevacizumab was considered inactive, signs of activity were noted in highly refractory patients. Our study confirmed that histology-specific phase II trials with targeted agents for MPNST are feasible and that predefined trial success can be dramatically altered based on choice of response criteria (the WHO versus RECIST). Our hope is that as more effective targeted combination therapies, which result in substantial tumor shrinkage, are discovered in preclinical models, translation to the clinic will also yield sustained objective tumor regression.

## Figures and Tables

**Table 1:** Patients' baseline characteristics.

Characteristic	All (*N* = 25)	NF1 MPNST (*N* = 17)	Sporadic MPNST (*N* = 8)
Median age at diagnosis, years (range)	37 (19, 81)	28 (19, 63)	61 (19, 81)
Female, *n* (%)	15 (60)	10 (58.8)	5 (62.5)
Asian, *n* (%)	1 (4)	1 (5.9)	0 (0)
Black or African American, *n* (%)	8 (32)	5 (29.4)	3 (37.5)
White, *n* (%)	16 (64)	11 (64.7)	5 (62.5)
Primary tumor location, *n* (%)			
Extremities	10 (40)	7 (41.3)	3 (37.5)
Trunk			
Chest wall	3 (12)	3 (17.6)	0 (0)
Spine	2 (8)	2 (11.8)	0 (0)
Mediastinal soft tissue	1 (4)	1 (5.9)	0 (0)
Pelvis	2 (8)	2 (11.8)	0 (0)
Retroperitoneum	1 (4)	0 (0)	1 (12.5)
Abdominal wall	1 (4)	1 (5.9)	0 (0)
Axilla	2 (8)	1 (5.9)	1 (12.5)
Parotid gland	1 (4)	0 (0)	1 (12.5)
Primary tumor resected, *n* (%)	20 (80)	13 (76.5)	7 (87.5)
Margins of surgical resection, *n* (%)			
R0: microscopic negative	9 (45)	7 (53.8)	2 (28.6)
R1: microscopic positive	4 (20)	2 (15.4)	2 (28.6)
R2: gross residual disease	5 (25)	3 (23.1)	2 (28.6)
If resected, did the primary tumor locally recur, *n* (%)	*N* = 20	*N* = 13	*N* = 7
No	5 (25)	2 (15.4)	3 (42.9)
Yes	15 (75)	11 (84.6)	4 (57.1)
Metastatic disease at diagnosis, *n* (%)	*N* = 21	*N* = 13	*N* = 8
No	18 (86)	12 (92.3)	6 (75)
Yes	3 (14)	1 (7.7)	2 (25)
Metastatic disease at enrollment, *n* (%)	*N* = 21	*N* = 13	*N* = 8
No	2 (10)	1 (7.7)	1 (12.5)
Prior chemotherapy, *n* (%)			
No	2 (8)	2 (11.8)	0 (0)
Yes	23 (92)	15 (88.2)	8 (100)
No. of prior treatment regimens			
Median (range)	2 (0, 5)	2 (0, 5)	2 (1, 4)
Patient had prior radiation, *N* (%)			
No	4 (16)	3 (17.6)	1 (12.5)
Yes	21 (84)	14 (82.4)	7 (87.5)
No. of prior radiation treatments			
Median (range)	1 (0, 3)	1 (0, 3)	1 (0, 2)
Patient had prior surgeries, *N* (%)			
No	2 (8)	1 (5.9)	1 (12.5)
Yes	23 (92)	16 (94.1)	7 (87.5)
No. of prior surgeries			
Median (range)	2 (0, 8)	2 (0, 8)	2 (0, 5)
ECOG at baseline, *N* (%)			
0	12 (48)	8 (47.1)	4 (50)
1	11 (44)	7 (41.2)	4 (50)
2	2 (8)	2 (11.8)	0 (0)

**Table 2:** Tumor response evaluation based on the WHO and RECIST criteria (stage 1: 15 patients; stage 2: 10 patients).

Response evaluation	Stage 1		Stage 2	
	Cycle 2 (*N* = 15)	Cycle 4 (*N* = 8)	Cycle 2 (*N* = 10)	Cycle 4 (*N* = 5)
WHO				
Partial response	1^*∗*^	0	0	0
Stable disease	7	1	5	2
Progressive disease	5	5	4	3
Others	2	2	1	0
RECIST				
Partial response	0	0	0	0
Stable disease	9	2	5	4
Progressive disease	4	4	4	1
Others	2	2	1	0

^*∗*^Partial response was unconfirmed and thus not counted as clinical benefit.

**Table 3:** Adverse events resulting in dose reduction (DR) or discontinuation (DC) of therapy.

Adverse events	*N*	Grade	Attribution		Intervention	
			Everolimus	Bevacizumab	Everolimus	Bevacizumab
Abdomen hemorrhage	1	5	−	+	DC	DC
Alanine aminotransferase	1	3	+	−	DC	DC
Proteinuria	1	2	−	+	DC	DC
Proteinuria^*∗*^	1	1	−	+	DC	DC
Neurologic decline^*∗*^	1	3	−	−	DC	DC
Back pain	1	2	−	−	DC	DC
Pain^*∗*^	1	4	−	−	DC	DC
Thromboembolism	1	3	−	−	None	DC
Fatigue	1	3	+	+	DR	None
Mucositis	2	2	+	−	DR	None
Mucositis	1	1	+	−	DR	None
Ear pain	1	1	+	−	DR	None

^*∗*^Off-treatment reason for these patients was disease progression.

## Data Availability

The data used to support the findings of this study are available from the corresponding author upon request.

## Supplementary Materials

Supplementary Materials All adverse events and adverse events with possible, probable, or definitive attribution to everolimus and/or bevacizumab that occurred in more than 1 patient.
