# Supplementary material for: Targeting Sporadic and Neurofibromatosis Type 1 (NF1) Related Refractory Malignant Peripheral Nerve Sheath Tumors (MPNST) in a Phase II Study of Everolimus in Combination with Bevacizumab (SARC016)
Source: Sarcoma. 2019 Jul 24;2019:7656747. doi: 10.1155/2019/7656747 (PMC6681622; doi:10.1155/2019/7656747)
Supplement: Supplementary Materials — All adverse events and adverse events with possible, probable, or definitive attribution to everolimus and/or bevacizumab that occurred in more than 1 patient. [file 7656747.f1.pdf]

Supplemental Table 1: All adverse events and adverse events with possible, probable, or definitive attribution to everolimus and/ or bevacizumab (highest grade-CTCAEv4- per patient) that occurred in more than 1 patient (4%). Total number of 25 patients (percent).

**Adverse events**	**Related adverse events**					**All adverse events**				
	**Grade 1**	**Grade 2**	**Grade 3**	**Grade 4**	**Grade 5**	**Grade 1**	**Grade 2**	**Grade 3**	**Grade 4**	**Grade 5**
**Blood and lymphatic system disorders**										
Anemia			1 (4)				1 (4)	2 (8)		
Platelet count decreased	3 (12)	1 (4)				3 (12)	1 (4)			
White blood cell decreased	2 (8)					2 (8)		1 (4)		
**Gastrointestinal disorders**										
Constipation	1 (4)	1 (4)				4 (16)	1 (4)			
Diarrhea	2 (8)					3 (12)		1 (4)		
Dyspepsia	1 (4)	1 (4)				1 (4)	1 (4)			
Mucositis oral	6 (24)	2 (8)	5 (20)			6 (24)	2 (8)	5 (20)		
Oral pain		1 (4)	1 (4)				1 (4)	1 (4)		
Nausea	4 (16)	3 (12)				5 (20)	3 (12)			
Vomiting	2 (8)	2 (8)	1 (4)			3 (12)	2 (8)	1 (4)		
**General disorders**										

SARC016 Manuscript: Supplementary Data Table

Adverse events	Related adverse events					All adverse events				
	Grade 1	Grade 2	Grade 3	Grade 4	Grade 5	Grade 1	Grade 2	Grade 3	Grade 4	Grade 5
Fever	1 (4)					2 (8)				
Fatigue	5 (20)	3 (12)	2 (8)			5 (20)	4 (16)	2 (8)		
**Investigations**										
Alanine aminotransferase increased		1 (4)	2 (8)				1 (4)	2 (8)		
Creatinine increased	2 (8)					2 (8)	1 (4)			
Weight loss	5 (20)	1 (4)	1 (4)			5 (20)	2 (8)	1 (4)		
**Metabolism and nutrition**										
Anorexia	2 (8)	4 (16)	1 (4)			2 (8)	4 (16)	1 (4)		
Cholesterol high	2 (8)	1 (4)				3 (12)	1 (4)			
Dehydration	1 (4)	1 (4)	1 (4)			1 (4)	2 (8)	1 (4)		
Hypertriglyceridemia	1 (4)	1 (4)		1 (4)		1 (4)	1 (4)		1 (4)	
Hypokalemia	1 (4)					1 (4)		2 (8)		
Hyponatremia			1 (4)					2 (8)		
Hypophosphatemia		1 (4)	3 (12)				1 (4)	4 (16)		
**Nervous system disorder**										

SARC016 Manuscript: Supplementary Data Table

Adverse events	Related adverse events					All adverse events				
	Grade 1	Grade 2	Grade 3	Grade 4	Grade 5	Grade 1	Grade 2	Grade 3	Grade 4	Grade 5
Headache	1 (4)	1 (4)				2 (8)	1 (4)			
**Pain**										
Pain (neck, back, chest wall, ear, flank)	3 (12)	1 (4)				6 (24)	4 (16)	1 (4)		
**Renal and urinary disorders**										
Proteinuria	1 (4)	3 (12)				1 (4)	3 (12)			
**Respiratory, thoracic, and mediastinal disorders**										
Epistaxis	3 (12)					3 (12)				
Nasal congestion	1 (4)		1 (4)			1 (4)		1 (4)		
Dyspnea	1 (4)	2 (8)				2 (8)	2 (8)			
Sore throat		2 (8)	1 (4)				2 (8)	1 (4)		
**Skin and subcutaneous tissue disorders**										
Dry skin	1 (4)					2 (8)				
**Vascular disorders**										

SARC016 Manuscript: Supplementary Data Table

**Adverse events**	**Related adverse events**					**All adverse events**				
	**Grade 1**	**Grade 2**	**Grade 3**	**Grade 4**	**Grade 5**	**Grade 1**	**Grade 2**	**Grade 3**	**Grade 4**	**Grade 5**
Hypertension	3 (12)	2 (8)	1 (4)			3 (12)	2 (8)	1 (4)		
